# Diet, Dermatology and Rotten Fish

**Published:** 1990-03

**Authors:** J. L. Burton

**Affiliations:** Department of Dermatology, Bristol Royal Infirmary, BS2 8HW


### Diet and Dermatology: Rotten Fish, Red Herrings and Essential Fatty Acids*

**J. L. Burton BSc, MD, FRCP**
Department of Dermatology, Bristol Royal Infirmary, BS2 8HW

Frederick Parkes Weber died in 1962 at the age of 99, having worked as a physician at the German hospital in Queen Square, London until the age of 80 (1). He published over 1000 papers and his name has of course been attached as an eponym to several diseases, notably the Sturge-Weber and the Osler-Weber-Rendu syndromes. He was fascinated by rare skin diseases, and some idea of his prodigious knowledge of the literature may be gained from the fact that on one occasion, when he stood up at the Royal Society of Medicine and admitted that he had never heard of the syndrome under discussion, there was tumultuous applause, acompanied by cheers and stamping of feet. It is a reflection of our increasing knowledge that the particular syndrome under discussion was Turner's syndrome, nowadays well-known to most medical students.

The dermatology textbooks which Weber would have read as a medical student in the 1880s were very keen on the idea that many dermatoses were caused by dietary factors (2). The great Jonathon Hutchinson of The London Hospital for example believed that leprosy was due to eating rotten fish, despite the fact that leprosy was endemic in several regions where fish was rarely eaten, and the lepra bacillus had been discovered some years previously, in 1873 (3).

Other physicians swallowed equally fishy stories. Radcliffe Crocker, of University College Hospital for example believed that atopic eczema was caused by irritation of the gastro-intestinal tract, especially by fermentable foods such as bread, beer and pastry. These gastric irritants were thought to produce reflex derangement of the nerve centres, with consequent dilatation of the skin capillaries (2).

Dermatologic gullibility was not confined to London however. Anderson of Glasgow felt that eczema was caused by a diet 'deficient in nutritive properties' (4), which most diets in Victorian Glasgow probably were, and according to Mrs Currie, still are. Eczema, he said, was 'a disease of the chlorotic, the rachitic, the scrofulous and the debilitated'. He quotes the case of a 15 month old child with atopic dermatitis, more or less given up for dead by the previous medical attendant, who was apparently cured within 5 weeks by the 'internal adminstration of codliver oil and iron alone'. Would betamethasone ointment have done as well?

That was the Glasgow view. Jamieson of Edinburgh, as we should expect, strongly disagreed. He postulated that eczema was caused by dietary changes consequent upon civilization such as the increased refining of flour (5). Refinement was not the only danger facing the Edinburgh bourgeoisie however . . . 'most harmful of all in predisposing to eczema is the abuse of tea, which by retarding tissue change leads to the accumulation of waste products in the system, and the imperfect removal of excrementitious matters already existent will be further complicated by the presence of the products of incomplete combustion'. One has to admire Jamieson's appropriately flatulent style.

A few years later Walker, also of Edinburgh, put the wind up even more Celtic kilts with this stern warning. 'Porridge insufficiently cooked, as it too often is out of Scotland, is undoubtedly as bad for patients with eczema as it is unpalatable to all' (6).

* (Based on part of the Parkes Weber Lecture, delivered at the Royal College of Physicians, London on Nov. 29th 1988.)

These views are not now universally accepted, but there is little doubt that some skin diseases can be directly caused by dietary factors (Table 1).

**Table 1**

**Dietary dermatoses**

A. *Dermatoses caused by diet*
1. Nutrient excess
   (a) Generalized, e.g. acanthosis nigricans due to obesity
   (b) Specific, e.g. orange palms due to hypercarotenaemia
2. Nutrient deficiency
   (a) Generalized, e.g. hair changes due to protein/calorie deprivation
   (b) Specific, e.g. perifollicular haemorrhage due to scurvy
3. Toxins
   (a) Food or drink, e.g. flushing from alcohol or red peppers
   (b) Natural contaminant, e.g. ergotism due to fungus
   (c) Chemical contaminant, e.g. porphyria turcica due to hexachlorobenzene
4. Allergy, e.g. urticaria
5. Idiosyncracy, e.g. alcohol/chlorpropamide flushing in diabetes

B. *Dermatoses prevented by diet*
   ? Prevention of skin cancer by anti-oxidants

C. *Modulation of pre-existing dermatoses*
   Suppression of cutaneous inflammation by essential fatty acids

### DIETARY DERMATOSES

### 1. Excessive calorie intake

Obesity is associated with increased sweating and a tendency to intertrigo of the body folds. Some obese patients also have acanthosis nigricans and a tendency to form skin tags around the neck. This is associated with insulin resistance and hyperinsulinaemia (7, 8). The skin changes may be due to binding of insulin to peptide growth factors which stimulate epidermal growth (9). It is possible that the same abnormality predisposes to both obesity and acanthosis nigricans, but the condition is probably not due entirely to an inherent cellular defect because the partial response of the insulin resistance to fasting suggests that an acquired defect related to obesity is also involved (10).

Acanthosis nigricans is also associated with lipodystrophic diabetes, and this type has been reported to respond to dietary treatment with fish oil supplements (11). This may however partly result from a beneficial effect of the fish oil on the diabetes (12).

### 2. Excess of a single food factor

Hypervitaminosis A causes diffuse alopecia, scaling of the skin, liver damage and increased intra-cranial pressure. This occurred in some of the early Arctic explorers who ate polar bear liver, which contains huge stores of vitamin A derived from the fish-seal food chain.

More commonly seen nowadays is hypercarotenaemia which causes orange discloration of the palms and soles. This occurs predominantly in slimmers on a low-calorie diet wth an excess of carrots and oranges (13).

**3. Chronic starvation**

Deficiency of protein and calories causes a scaly rash with a tendency for the hair to become dry and sparse (4). Kwashiorkor produces a characteristic reddish discoloration of black hair, and the scaling of the skin often takes on the glazed appearence of adherent enamel flakes. The 'flag sign', with alternating dark and light bands in the individual hairs, is due to intermittent protein deficiency, and measurement of various parameters in the hair shaft has been suggested as a useful measure of nutritional status, particularly for proteins (15).

Patients with anorexia nervosa develop dry skin, lanugo-like body hair, thinning of the scalp hair and brittle nails due to starvation (16).

**4. Specific deficiencies**

These provide the classical examples of dietary dermatoses. The cutaneous manifestations of deficiencies of the various vitamins, iron, zinc etc are well-known, and have been reviewed elsewhere (17–19).

Many of these deficiency states have increased our knowledge of intermediary metabolism. Animals fed only on dried eggs, for example develop alopecia and a scaly dermatitis which resembles seborrhoeic dermatitis (20, 21). Biotin deficiency in man also causes dermatitis (22) and in the period of austerity of the 1940s some babies with severe seborrhoeic dermatitis apparently responded well to biotin therapy (23).

Overt biotin deficiency is rare, because the dietary requirements are so small that a biotin deficient diet is usually incompatible with life. Occasional cases of biotin deficiency with alopecia and dermatitis after prolonged parenteral feeding have been reported (24) and one otherwise healthy male eccentric managed to become biotin deficient by living largely on wine with a raw egg in each glass (25). Egg whites contain avidin, a glycoprotein which binds to any small quantity of biotin in the diet and inactivates it.

Biotin is necessary as a co-factor for the activity of several carboxylases, and multiple carboxylase deficiency can be inherited as a genetic disorder (26). Children with this defect are bald, but they grow hair if they are given large doses of biotin. The alopecia of biotin deficiency may respond to dietary fatty acids (27). Carboxylases are required for normal keratin formation and veterinary surgeons nowadays give biotin supplements to help pigs and horses to make better hooves (28). Whether biotin supplements will help brittle nails remains to be seen, but essential fatty acid supplements have been claimed to improve the brittle nails of Sjogren's syndrome (29).

**5. Toxins**

Dermatological toxins come from several dietary sources. The ingested food or drink may be inherently toxic, or there might be a natural contaminant such as a fungus, or some added chemical, such as an adulterant.

Alcohol is a cutaneous vasodilator, and some races such as Mongoloids are particularly susceptible to alcohol-induced flushing (30). Chillies and red peppers also produce flushing and sweating by a pharmacological action (31).

Ergotism, characterized by severe Raynaud's phenomenon leading to digital gangrene, provides the classical example of skin changes due to ingestion of a fungal contaminant, and a dermatosis due to a chemical contaminant occurred in Turkey when wheat which had been treated with a fungicide, hexachlorobenzene, caused many cases of porphyria cutanea tarda (32). This was characterized by photosensitivity, fragile skin, scleroderma, hypertrichosis and painless arthritis.

**6. Allergic reactions**

Urticaria is often due to immediate hypersensitivity to an ingested food allergen, but contact urticaria due to food is less common (33). In this condition the urticaria often occurs around the lips, often in a child with atopic eczema, and may be accompanied by vomiting of the ingested allergen.

A more complicated allergic reaction is the gluten enteropathy which is associated with dermatitis herpetiformis. If gluten is strictly avoided for many months and the bowel completely recovers, the dapsone requirements decrease, and the skin disease may even go into complete remission (34).

**7. Idiosyncratic reactions**

Individual susceptibility to a dietary factor may be commoner than is generally suspected. Some cases may be due to interactions with systemic medication, e.g. the severe alcohol-induced flushing seen in some patients who take chlorpropamide (35–37), or the reaction to cheese or pickles in patients taking mono-amine oxidase inhibitors. Other idiosyncratic reactions may be due to an anatomic abnormality, as in the flushing which accompanies the post-gastrectomy dumping syndrome.

Some idiosyncratic reactions such as the urticaria, flushing and bronchospasm which can follow the ingestion of metabisulphites used to keep fruit and vegetable fresh may be mediated by neural activity, since tests for allergy are negative and the adverse reaction is blocked by atropine (38). The Chinese restaurant syndrome (Kwok's quease), in which similar symptoms follow the ingestion of sodium glutamate, seems to be mediated by transient release of acetylcholine-like compounds (39–40).

### DIETARY PREVENTION OF SKIN DISEASE

Some dietary factors might help to prevent the development of cutaneous disease. Anti-oxidants such as ascorbic acid in large doses can reduce the incidence of ultra-violet irradiation-produced skin cancers in animals (41, 42). This effect is thought to be mediated by the inhibition of production of cholesterol alpha-oxide, a carcinogenic steroid which is produced by the ultra-violet irradiation of skin.

### DIETARY MODULATION OF DERMATOSES

'Should I be on a special diet, doctor?' is probably second only to 'Could it be due to hot blood?' as a question guaranteed to provoke a dermatological sigh. The history of dermatology is littered with the corpses of discarded dietary hypotheses, some of which still stir feebly. Several studies have shown that chocolate does not affect the course of acne for example (43, 44), but despite that, many intelligent patients, some of whom are doctors, claim that chocolate or some other dietary factor, makes their acne worse. Some of these cases may be idiosyncratic reactions.

Another minefield is the use of exclusion diets for the treatment of atopic eczema (45). Women's magazines generally seem to have more success with this than dermatologists, and a recent survey showed that more than 70% of children with atopic eczema had excluded eggs, dairy products etc from their diet before attendance at hospital, but less than 10% had noticed any benefit (46).

Dietary fat supplements were widely used in the 1930s and 1940s to treat atopic eczema, which was in those days fairly intractable, but they fell into desuetude when topical steroids were introduced. Several groups have recently shown that oral evening primrose oil, which contains fatty acids, has a beneficial effect in eczema (47–50). Some atopic patients have low levels of essential fatty acid metabolites such as dihomogammalinolenic acid in their plasma (51), and evening primrose oil is particularly rich in the precursor of this compound, gamma-linolenic acid. An analysis of some recent large trials has shown that there is a correlation between the clinical improvement and the increase in essential fatty acid metabolite levels after treatment with the evening primrose oil (50). The exact mechanism is not yet known, although it seems very likely from animal experiments that dietary essential fatty acids modulate the metabolism of the eicosanoids (prostaglandins, leukotrienes and thromboxanes) to reduce inflammation and itching. Essential fatty acids also have a direct structural role in the epidermal barrier layer, and it is possible that a defect in the water barrier layer could account for the dry skin which is such a prominent feature in many atopic eczema patients. These ideas have been discussed in more detail elsewhere (52, 53).

### REFERENCES

1. Obituary: Frederick Parkes Weber. (1962) *Brit. Med. J.* **i**, 1630–1.
2. BURTON, J. L. (1988) Diet and dermatology in 1888: the influence of H Radcliffe Crocker. *Brit. J. Derm.* **119**, 471–8.
3. RUSSELL, B. F. (1969) Dermatology at the London Hospital. *Brit. J. Derm.* **81**, 780–93.
4. ANDERSON, T. M. (1894) A Treatise on Diseases of the Skin, with special reference to their diagnosis and treatment (2nd edn). Griffin: London. p. 154.
5. JAMIESON W. A. (1869) Diseases of the Skin; A Manual for Practitioners and Students (2nd edn). Young J. Pentland: Edinburgh. p. 227.
6. WALKER, N. (1916) An Introduction to Dermatology (6th edn). Green W: Edinburgh. p. 118.
7. KAHN, C. R., FLIER, J. S., BAR, R. S. *et al.* (1976) The syndromes of insulin resistance and acanthosis nigricans: Insulin receptor disorders in man. *New Eng. J. Med.* **294**, 739–45.
8. FLIER, J. S. (1985) Metabolic importance of acanthosis nigricans. *Arch. Dermatol.* **121**, 193–4.
9. BLUNDELL, T. L., BEDARKIAN, S., HUMBREL, R. E. (1983) Tertiary structures, receptor binding and antigenicity of insulin-like growth factors. *Fed. Proc.* **42**, 2592–7.
10. PETERS, E. T., STUART, C. A., PRINCE, M. J. (1986) Acanthosis nigricans and obesity: Acquired and intrinsic defects in insulin action *Metabolism* **35**, 807–13.
11. SHERERTZ, E. F. (1988) Improved acanthosis nigricans with lipodystrophic diabetes during dietary fish oil supplementation. *Arch. Dermatol.* **124**, 1094–6.
12. CUNNANE, S. C., MANKU, M. S., HORROBIN, D. F. (1985) Abnormal essential fatty acid composition of tissue lipids in genetically diabetic mice is partially corrected by dietary linoleic acid and gamma-linolenic acids. *Brit. J. Nutr.* **53**, 449–58.
13. BILIMORIA, S., KECKES, K. *et al.* (1979) Hypercarotenaemia in weight-watchers. *Clin. Exp. Dermatol.* **4**, 331–4.
14. MACLAREN, D. S. (1987) Skin in protein energy malnutrition. *Arch. Dermat.* **123**, 1674–6.
15. SIMS, R. T. (1968) The measurement of hair growth as an index of protein synthesis in malnutrition. *Brit. J. Nutr.* **22**, 229.
16. GUPTA, M. A., GUPTA, A. K., HABERMAN, H. F. (1987) Dermatologic signs in anorexia nervosa and bulimia nervosa. *Arch. Dermatol.* **123**, 1386–90.
17. BARTHELEMY, H. *et al.* (1986) Skin and mucosal deficiencies in vitamin deficiency. *J. Am. Acad. Derm.* **15**, 1263–74.
18. ROE, D. (1986) Nutrition and the Skin. Contemporary Issues in Clinical Nutrition. Vol. 10. Liss: New York.
19. FRAKER, B. J., JARDIEU, P., COOK, J. (1987) Zinc deficiency and immune functions. *Arch. Dermatol.* **123**, 1699–701.
20. BOAS, M. A. (1927) The effect of desiccation upon the nutritive properties of egg white. *Biochem. J.* **21**, 712–24.
21. WAISMAN, H. A., MacCOLL, K. B., ELVEHJEM, C. A. (1945) Acute and chronic biotin deficiencies in the monkey. *J. Nutrition* **29**, 1–10.
22. SYDENSTRICKER, V. T., SINGAL, S. A., BRIGGS, A. P., De VAUGHN, M. B. (1942) Preliminary observations on egg-white injury in man and its cure with a biotin concentrate. *Science* **95**, 176.
23. NISENSON, A. (1957) Seborrhoeic dermatitis of infants and Leiner's disease: a biotin deficiency. *J. Pediatr.* **51**, 537–70.
24. MOCK, D. M., DeLORIMER, A. A., LIEBMAN, W. M., SWEETMAN, L., BAKER, H. (1981) Biotin deficiency; an unusual complication of parenteral alimentation. *New Eng. J. Med.* **304**, 820–3.
25. WILLIAMS, R. H. (1943) Clinical biotin deficiency. *New Eng. J. Med.* **228**, 247–55.
26. NYHAN, W. L. (1987) Inborn errors of biotin metabolism. *Arch. Dermatol.* **123**, 1696–8A.
27. MUNNICH, A., SANDUBRAY, J. M., COUDE, F. X. *et al.* (1980) Fatty acid responsive alopecia in multiple carboxylase deficiency. *Lancet* **i**, 1080–1.
28. COMBEN, N., CLARK, R. J., SUTHERLAND, D. J. B. (1984) Clinical observations on the response of equine hoof defects to dietary supplementation with biotin. *Veterinary Record* **115**, 642–5.
29. CAMPBELL, A. J., McEWEN, G. C. (1981) Treatment of brittle nails and dry eyes. *Brit. J. Derm.* **105**, 113.
30. WOLFF, P. H. (1972) Ethnic differences in alcohol sensitivity. *Science* **175**, 449–50.
31. WILKIN, J. K. (1983) Flushing reactions. In 'Recent Advances in Dermatology'. Vol. 6. Ed. R. H. Champion. Churchill Livingstone: Edinburgh. pp. 158–87.
32. CRIPPS, D. J. (1980) Porphyria turcica, twenty years after hexachlorobenzene intoxication. *Arch. Dermatol.* **116**, 46–50.
33. HANNUKSELA, M. (1986) Contact urticaria from foods. In: Nutrition and the Skin (ed. by D. Roe). Liss: New York.
34. HALL, R. P. (1987) Dietary management of dermatitis herpetiformis. *Arch. Dermatol.* **123**, 1378–80.
35. BARNETT, A. H. PYKE, D. A. (1980) Chlorpropamide-alcohol induced flushing and large vessel disease in non-insulin dependent diabetes mellitus. *Brit. Med. J.* **2**, 261–2.
36. BARNETT, A. H., SPILIOPOULOS, A. J., PYKE, D. A. (1980) Blockade of chlorpropamide-alcohol flushing by indomethacin suggests an association between prostaglandins and diabetic vascular complications. *Lancet*, **ii**, 164–6.
37. KOBBERLING, J., WEBER, M. (1980) Facial flush after chlorpropamide-alcohol and enkephalin. *Lancet* **i**, 538–9.
38. SETTIPANE, G. (1984) Adverse reactions to sulfite in drugs and foods. *J. Am. Acad. Derm.* **10**, 1077–80.
39. GHADIMI, H., KUMAR, S., ABACI, F. (1971) Studies on monosodium glutamate ingestion: 1. Biochemical explanation of the Chinese Restaurant syndrome. *Biochem. Med.* **5**, 447–56.
40. REIF-LEHRER, L. (1976) Possible significance of adverse reactions to glutamate in humans. *Fed. Proc.* **35**, 2205–11.
41. DUNHAM, W. B. *et al.* (1982) Effects of intake of L-ascorbic acid on the incidence of dermal neoplasms induced in mice by ultra-violet light. *Proc. Nat. Acad. Sci.* **79**, 7532–6.
42. BLACK, H. (1974) Dietary antioxidants (ascorbic acid, DL-alpha tocopherol and glutathione) reduce carcinogenic effects of ultraviolet irradiation in albino hairless mice. *Chem. Path. Pharmacol.* **7**, 783.
43. PLEWIG, G., KLIGMAN, A. L. (1975) Acne morphogenesis and treatment. Springer-Verlag: Berlin 1975, pp. 270–1.
44. FRIES, J. H. (1978) Chocolate – a review of published allergic and other deleterious effects, real and presumed. *Ann. Allergy* **41**, 195–207.
45. THODY, A. J., FRIEDMANN, P. S. (1986) Scientific Basis of Dermatology – a physiological approach. Churchill Livingstone: Edinburgh. pp. 321–9.
46. WEBBER, S. A., GRAHAM-BROWN, R. A. C. *et al.* (1988) Dietary manipulation in atopic dermatitis. *Brit. J. Derm.* **119**, 15 (Abstract).
47. WRIGHT, S., BURTON, J. L. (1982) Oral evening-primrose-seed oil improves atopic eczema. *Lancet* **ii**, 1120–2.
48. SCHALIN-KARRILA, M., MATTILA, L., JANSEN, C. T., UOTILA, P. (1987) Evening primrose oil in the treatment of atopic eczema: effect on clinical status, plasma phospholipid fatty acids and circulating blood prostaglandins. *Brit. J. Dermatol.* **117**, 11–19.
49. SUGAI, T. (1987) Clinical evaluation of oral evening primrose oil in atopic patients. *Skin Research* **29**, 330–8.
50. MORSE, P. F., HORROBIN, D. F. *et al.* (1989) Meta-analysis of placebo-controlled studies of the efficacy of Epogam in the treatment of atopic eczema: Relationship between plasma essential fatty acid changes and clinical response. *Brit. J. Derm.* **121**, 75–90.
51. MANKU, M. S., HORROBIN, D. F. *et al.* (1982) Reduced levels of prostaglandin precursors in the blood of atopic patients: Defective delta-6 desaturase function as a biochemical basis for atopy. *Prostaglandins, Leukotrienes and Medicine* **9**, 1–13.
52. BURTON, J. L. (1989) Dietary fatty acids and inflammatory skin disease. *Lancet*, **i**, 27–31.
53. BURTON, J. L. (1989) 'Essential fatty acids and the skin'. In Recent Advances in Dermatology, edited by R. Champion and R. Pye. Churchill Livingstone: Edinburgh (in press).

